# Current Progress in Understanding and Recovering the Wheat Genes Lost in Evolution and Domestication

**DOI:** 10.3390/ijms21165836

**Published:** 2020-08-14

**Authors:** Shanjida Rahman, Shahidul Islam, Zitong Yu, Maoyun She, Eviatar Nevo, Wujun Ma

**Affiliations:** 1 State Agricultural Biotechnology Centre, College of Science, Health, Engineering and Education, Murdoch University, Perth, WA 6150, Australia; shanjida.rahman@murdoch.edu.au (S.R.); s.islam@murdoch.edu.au (S.I.); zitongyu@outlook.com (Z.Y.); m.she@murdoch.edu.au (M.S.); 2 Institute of Evolution, University of Haifa, Haifa 31905, Israel; nevo@evo.haifa.ac.il

**Keywords:** gene modification, wild emmer wheat, evolution and domestication, novel genes, trait enhancement

## Abstract

The modern cultivated wheat has passed a long evolution involving origin of wild emmer (WEM), development of cultivated emmer, formation of spelt wheat and finally establishment of modern bread wheat and durum wheat. During this evolutionary process, rapid alterations and sporadic changes in wheat genome took place, due to hybridization, polyploidization, domestication, and mutation. This has resulted in some modifications and a high level of gene loss. As a result, the modern cultivated wheat does not contain all genes of their progenitors. These lost genes are novel for modern wheat improvement. Exploring wild progenitor for genetic variation of important traits is directly beneficial for wheat breeding. WEM wheat (*Triticum dicoccoides*) is a great genetic resource with huge diversity for traits. Few genes and quantitative trait loci (QTL) for agronomic, quantitative, biotic and abiotic stress-related traits have already been mapped from WEM. This resource can be utilized for modern wheat improvement by integrating identified genes or QTLs through breeding.

## 1. Introduction

With the beginning of agriculture in the Neolithic period, plants having symbiotic relation with human experienced evolutionary process which ultimately promoted human cultural development and human civilization [1]. Some of those (rice, wheat and maize) are now considered staple foods and feed a large proportion of the world’s population. Thus, a crop’s evolutionary process can be used as genetic and ecological models to evolutionary biologists for studying human–plant interactions [2]. A better understanding about the origin of crops, which remained unchanged in ploidy level during domestication from wild ancestors (such as rice and maize), has already been obtained through the advancement of modern molecular biology [3]. For many other crops, the origin, domestication, and diversification of many genes are largely unexplored. The evolution of wheat went through a long and multiple processes, including natural hybridization, polyploidization, domestication, and mutation that took place for more than 300,000 years, making it be a distinct model plant for evolutionary study [1].

At the early stage of evolution, it was difficult for new species to survive as a combination of different genome enveloped within one nucleus and followed by chromosome doubling resulted in severe genetic stress [4,5]. To cope up with stress, they had to face several challenges, such as rapid differentiation of homologous chromosomes for preventing inter-genomic pairing or securing intra-genomic pairing at meiosis and arranging inter-genomic genetic expression for harmonic coexistence [6]. These challenges are meet up through immediate genomic changes, including chromosome re-patterning, chromatin re-modelling, and molecular alteration [5,7]. Additionally, numerous morphological and physiological changes occurred during domestication which termed as ‘domestication syndrome’, including changes in seed dispersal mode, in plant architecture, increase in kernel size, loss of seed dormancy and change in nutrient content [8]. Even some genes get lost forever during evolution. Natural wheat and related allopolyploids have 2–10% less DNA than the sum of their parents which indicates elimination of DNA during evolution [9,10]. Another reason behind rapid alteration and genomic change is using different breeding method extensively. Particularly after World War II, the intensive breeding program was performed, focusing mainly on high yield. As a result, the gene pool had been narrowed down gradually, due to the enormous erosion of indigenous genetic resources [11].

For wheat improvement, adaptive genetic resources of wild progenitors and relatives can be utilized as they have enriched diversity and many beneficial traits. Exploring wild progenitor, such as wild emmer (WEM) wheat will be useful for wheat breeding and to observe wheat evolutionary changes. WEM was one of the basic plants in Neolithic agriculture, domestication of which was a key factor for the initiation of agriculture [12,13]. Landraces of WEM have huge gene pool that consists of a rich diversity for many important agronomic, qualitative biotic stress, and abiotic stress-related traits [11,14,15,16]. Many of these genes did not enter hexaploidy wheat, thus, are considered as lost genes through evolution. This manuscript will focus on gene flow, and dynamics through genomic and morphological changes occurred during the wheat evolutionary process and different approaches recovering the lost genes in WEM for modern wheat improvement.

## 2. Evolution of Wheat

**Figure 1:**
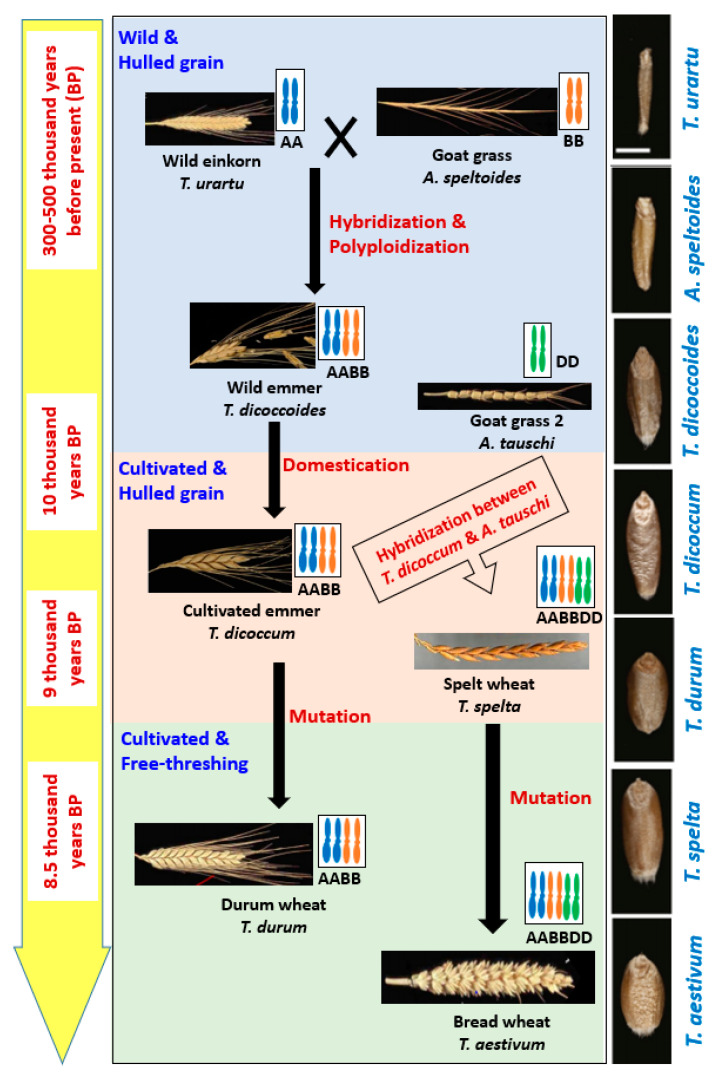
The central flow chart shows the evolution of wheat through hybridization, allopolyploidization, domestication and mutation along with modification in spike size and spike threshability. Left side yellow colored bar indicates the approximate time of those events happened, and right side black colored bar shows the gradual changes in grain size and shape during evolution.

Wheat belongs to the genus *Triticum* which includes six species: *Triticum monococcum* (AA); *Triticum urartu* Tumanian ex Gandilyan (AA); *Triticum turgidum* L. (AABB); *Triticum timopheevii* Zhuk. (AAGG); *Triticum aestivum* (AABBDD); and *Triticum zhukovskyi* Menabde and Ericz. (AAAAGG), which can be grouped into three categories: (i) Monococcum (2n = 2x), (ii) Dicoccoidiea (2n = 4x) and (iii) Triticum (2n = 6x). The reason behind these diversified species is an evolutionary process which is truly a very complex and long process that started at prehistoric Stone Age [2,17] (Figure 1).

WEM wheat (*Triticum dicoccoides*) was produced through hybridization between wild diploid wheat (*T. urartu*, 2n = 2x = 14, genome AA) and Goat Grass 1 (*Aegilops speltoides*, 2n = 2x = 14, genome BB) around 0.3–0.5 million years ago [17,18,19]. Two probable ways of developing WEM were: (i) Interspecific hybridization and then chromosome doubling in the sterile hybrid and (ii) crossing of unreduced parental gametes forming tetraploid wheat [10,20]. Cultivated emmer wheat (*T. turgidum* spp. *Dicoccum*) evolved gradually through subconscious selection from WEM by ancient people, particularly by hunter-gatherers, around 10,000 years ago in the Fertile Crescent region. The oldest evidence of cultivated emmer was observed in Tell Aswad, Syria, around 9500 years ago [2]. Moreover, some other evidence was also found in several other pre-pottery Neolithic sites in the Fertile Crescent region [2]. This region (Fertile Crescent) still has some WEM that can be divided into two groups, southern (grown in Israel, Lebanon, Palestine, and southwestern Syria) population and northern (grown in Iraq, Iran and Turkey) population [21]. Two ideas were found describing domestication of WEM: (a) Domestication was started in the northern region of the fertile crescent and instant spread to the south or vice-versa, (b) domestication occurred in both northern and southern part independently [22]. However, later, some other archaeological evidence strongly suggested independent domestication and cultivation of WEM in multiple sites [2].

Domesticated emmer hybridized spontaneously later with another wild genotype called Goat Grass 2 (*Aegilops tauschii*, 2n = 2x = 14, genome DD) and produced hexaploid spelt (*Triticum spelta*, 2n = 6x = 48, genome AABBDD) wheat around 9000 years ago [23,24]. Both cultivated emmer and spelt wheat were characterized with hulled grain, i.e., spikelet as the threshing product (Figure 1).

Free-threshing durum (*Triticum durum*, 2n = 4x = 28, genome AABB) and bread wheat (*T. aestivum*, 2n = 6x = 42, genome AABBDD) were originated from enclosed cultivated emmer and spelt wheat, respectively, around 8500 years ago through natural mutation [2]. Clearly, emmer wheat played the central role of wheat evolution. Different opinions also found in the literature which explained the origin of bread wheat as a crossing product between (i) the hulled cultivated emmer (*T. dicoccum*), (ii) free-threshing the *T. durum*, or (iii) the free-threshing *Triticum parvicoccum* with the *Ae. Tauschii* [23,24]. However, the crossing took place probably 9000 years ago in south or west of the Caspian Sea, just after spreading of emmer wheat cultivation from Fertile Crescent into the natural habitat of *Ae tauschii* [25,26]. These studies further strengthened the central role status of emmer wheat in the evolutionary process.

## 3. Changes in the Wheat Genome during Evolution

### 3.1. Genomic Changes through Domestication

Generally, domestication is a selection process that provides the increased adaptation and economic viability of the plants to be cultivated in a particular environmental condition [27]. It is assumed that the first domestication of crop species started by humans 10,000 years ago [22,28]. The initial selection of wild plants as potential crops was the first step in the foundation of agriculture. However, plant selection under domestication is being continued since the Neolithic period through to plant breeding of today [29]. Through the domestication process, plants go through a suite of complex morphological, physiological, and genetic changes [30].

According to the history of wheat evolution, only wild einkorn and WEM wheats went through the early domestication selection [1]. Einkorn, *T. monococcum* is a diploid wheat, which was domesticated from the wild progenitor species *T. boeoticum* in the Fertile Crescent. Later on, it was gradually replaced by tetraploid and hexaploid wheats during the last 5000 years, approximately. Einkorn has never been involved in the evolution of hexaploid bread wheat or tetraploid durum wheat. The wild diploid *Triticum* species, which was the progenitor of hexaploid wheat and played an essential role in wheat evolution in *T. urartu* (AA) [28]. The tetraploid wheat species *T. dicoccoides* known as WEM naturally had been grown all over the Fertile Crescent. The early wheat growers domesticated WEM, and thus, cultivated emmer (*T. dicoccum*, AABB) was introduced. For a millennium or more since its domestication, emmer wheat was still growing with WEM in a complex cropping system in many Levantine sites. Thus, the genes (for example, non-brittleness gene) were transferred through spontaneous and uncontrolled hybridizations. As a result, the domesticated emmer wheat has appeared as polymorphic populations [22].

Generally, domestication aims with the elimination of undesired or even deleterious alleles, but almost in every case it also reflects an erosion of alleles valuable for plant improvement and future demands of producers and consumers [31]. It has been well documented that substantial genetic erosion occurred through the domestication process of wheat and that erosion was further reinforced during modern breeding processes [32,33,34]. Consequently, loss of diversity, selective sweeps and adaptive diversification have occurred that caused considerable genetic modification [27]. The development in molecular marker and quantitative trait loci (QTL) analysis techniques enabled to characterize those genetic losses or modifications. For example, nucleotide diversity at 21 gene loci was analyzed in wild, domesticated, cultivated durum and bread wheats, and revealed that diversity was reduced in cultivated forms during domestication by 69% in bread wheat and 84% in durum wheat [35].

The most significant effect of domestication is that genetic diversity has been reduced and is being continued through the modern plant breeding. The occurrence of genetic narrow down essentially has reduced the efficiency of crop improvement [36]. Wheat domestication by the early farmers eventually resulted in landrace cultivars (LCs) adapted to specific conditions of their habitats. At the advancement of modern plant breeding during the last century, most of the traditional LCs were continually replaced by modern wheat cultivars (MWCs) [37]. As the MWCs were bred from a few LCs they contain less genetic diversity than traditional LCs [36]. Growing wheat of such a narrow genetic diversity accelerates the risk of genetic vulnerability to the adverse condition. The risk has been further raised up due to the spontaneous mutations of a number of major insect and pathogens and the impulsive changes in environmental conditions. These might bring stresses in a new dimension that the present wheat cultivars could not cope with, and therefore, could lead to severe crop losses. During the second half of the last century number of such kind of severe crop loss had been evident. For examples, severe epidemics of shoot fly (*Atherigona spp.*) and kernel bunt (*Tilletia indica*); the outbreak of the southern corn leaf blight in the 1970s, and more recently, the outbreak of wheat blast in Bangladesh and northern India [36,38].

The genetic basis of the domestication syndrome in wheat has been extensively studied which revealed that the loss of genetic diversity in spring bread wheat occurred during (i) its domestication, (ii) the change from traditional landrace cultivars (LCs) to modern breeding varieties, and (iii) 50 years of international breeding [36]. Considerable loss of genetic diversity was observed at the early periodic domestication, and during the time of LCs to the elite breeding germplasm. It has also been evident that wheat’s genetic diversity was narrowed down more robustly during the time between 1950 and 1989. However, genetic diversity showed an uprising trend starting from 1990 indicating that breeders have experienced the consequence of narrowing down genetic diversity in the modern breeding and subsequently started to increase the genetic diversity through the introgression of novel materials. The LCs and *T. tauschii* contain numerous unique alleles that were absent in modern spring bread wheat cultivars [36].

It has been considered that, at the very beginning of the domestication process, the major domestication trait was the seed dispersal mode [8]. Certainly plants with reduced spikelet shattering at maturity had been domesticated, which was considered as a key feature in preventing natural yield losses [8]. In addition to the yield, the other major domestication-related traits include glume reduction (easier threshing), plant architecture (plant height, tiller numbers etc.), ear and kernel size, seed dormancy [39]. Later on, with the improvement of grain analytical process, the grain protein and mineral concentrations, as well as carbohydrate content, also became major selection attributes (Table 1). Domestication has genetically not only transformed the brittle rachis, tenacious glume and non-free threshability, but also modified numbers of other traits [8]. Meanwhile, breeding and selection had a different impact on different wheat genomes. For example, a greater number of genes related to those domestication traits are found on the A and B genomes [40]. Differential loss has been found that supports greater gene loss in the A and B genomes compared with the D [41].

Reduction in diversity caused by intensive selection can be counterbalanced by introgression of novel germplasm. The best strategy for wheat improvement is to utilize the adaptive genetic resources of the wild progenitors, wild emmer (WEM, *T. dicoccoides*) and other wheat relatives [15,34,49,50].

### 3.2. Genomic Changes through Polyploidization

During the polyploidization of wheat, rapid alteration and several genomic changes occurred in nature. Such phenomena can be divided into two groups: (i) Revolutionary changes, and (ii) evolutionary changes. Revolutionary changes took place rapidly, during or just immediate after allopolyploidization and within a few generations, whereas evolutionary changes happened throughout the evolutionary lane for hundreds to thousands of generations and accelerated by polyploidy [10]. These changes can be of various types, including the elimination of both low-copy and high-copy DNA sequences, intergenomic disruption of DNA sequences, DNA methylation, deletion of rRNA, gene loss, suppression or activation of gene, chromatin modification and remodeling, heterochromatinization, sub-functionalization, and neo-functionalization [51]. These changes are directly or indirectly influenced by allopolyploidization. Besides, hybridizations that occurred during the evolution of wheat also resulted in some significant genetic changes as this is a very common outcome of the process. For examples, in the crossing product (hybrid) of *Aegilops sharonensis* and *Aegilops umbellulate*, 14% loci from *Ae. Sharonensis* and 0.5% loci from *Ae. Umbellulate* were lost; whereas, in the case of a cross between *Ae. Sharonensis* and *T. monococcum*, many sequences from *T. monococcum* were doubled in hybrid compared to another parent [52]. However, it is evident that evolution results in several genomic changes. Some examples are given below in more details.

**Figure 2:**
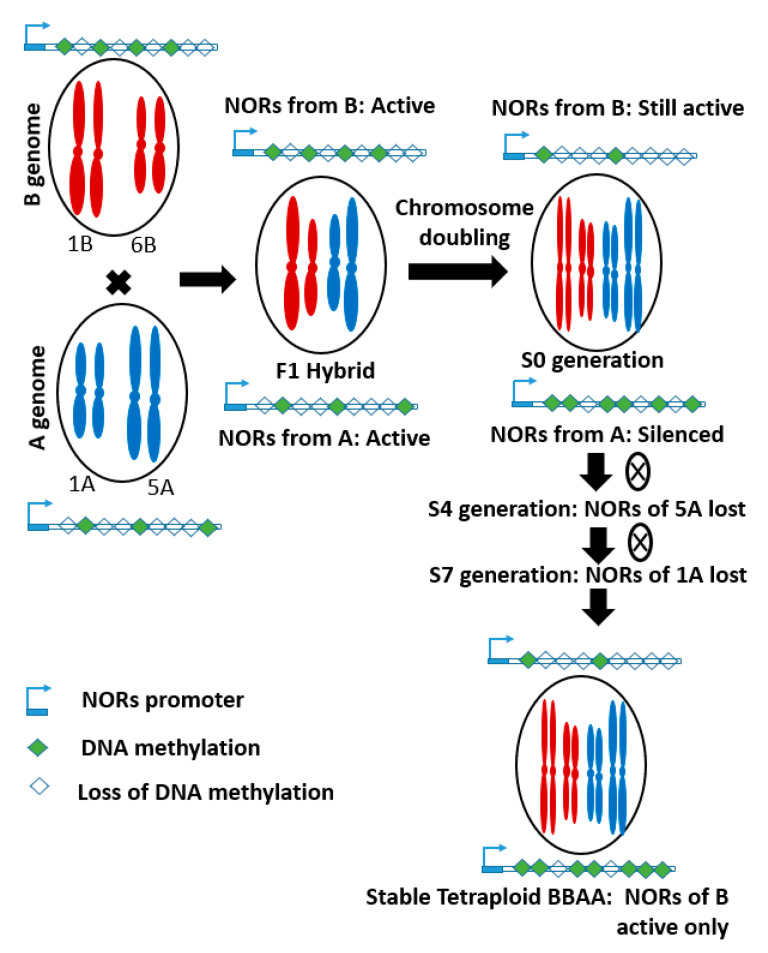
Schematic diagram showing the loss of Nucleolus organizing regions (NORs) from A genome, due to increased DNA methylation during the evolution process. S4 and S7 mean fourth and seventh generation of selfing (adopted from Guo and Han (2014) [7]).

Nucleolus organizing regions (NORs), also named as ribosomal DNA (rDNA) loci are present on different chromosomes (1A, 5A, 1B, 6B, and 5D) of diploid wheat [53]. This gene is composed of long tandem repeats that clustered on the chromosome and translated into important components of the chromosome [7]. Its activity is associated with the size of the intergenic regulatory region and the status of cytosine methylation [54,55]. However, NORs from the A genome are largely lost during the evolution of synthetic tetraploid wheat, due to asymmetric transcription and epigenetic modifications during polyploidization (Figure 2). In hybrids, NORs from both parents were expressed. However, after chromosome doubling, it became silenced in one parent (A genome), due to increased DNA methylation. In this process, a pair of NOR on the 5A chromosome were deleted first, the gradual elimination of another pair from the 1A chromosome, resulted in complete loss of NORs from the A genome by S7 generation. Therefore, in stable synthetic tetraploid wheat, rDNA from only the B genome was present. In the case of bread wheat, the rDNA loci form both A and D genomes were largely eliminated during evolution [7]. Additionally, genome wide transcription analysis revealed that gene expression in synthetic bread wheat is parentally dominant and only one of the parental genomes determines morphological traits and ecological adaptations [46,51,54,56].

Chromosome-specific sequences (CSSs) occur in only one homologous chromosome pair, i.e., 1A and 1A. These types of sequences were present in all diploid species. However, after polyploidization, CSSs from one genome were eliminated immediately or after some generations. As a consequence, in hexaploid or tetraploid, these sequences occur only in one homologous pair but absent from the homeologous chromosome [57]. Meanwhile, allopolyploidization results in rapid non-random deletion of specific non-coding, low-copy and high-copy DNA [58]. Again, sometimes some genes of the A and B genomes get suppressed upon adding of D genome. As results, they expressed in tetraploid AABB genome, but not in hexaploid AABBDD genome [10]. This is called intergenomic suppression. For example, the rust resistance gene(s) present in the A or B genome was suppressed by a gene present in the long arm of chromosome 7D [59].

### 3.3. Genomic Changes through Natural Mutation

Through the centuries, natural mutation resulted in significant changes in the genomic structures of wheat, which contributed substantially in the genetic evolutionary process of wheat. In general, mutation generated new alleles, while recombination created novel allele combinations. Accumulation of new mutations in older polyploid species, such as WEM, results in increased diversity and more uniform distribution across the genome [60]. For example, Genetic studies revealed that two recessive alleles at two major loci (*Br-A1* and *Br-B1*) controlling non-brittle rachis raised through mutation during domestication [61]. One of the most important genomic changes is the evolution of free-threshing wheat as a result of several major and minor mutation events. A single major gene *Q* on chromosome 5AL is responsible for free-threshing of modern bread and durum wheat, whereas the recessive *q* allele is for non-free-threshing wild wheats [1]. A recent study showed that the *Q* allele arose from *q* allele through a gain of function mutation [62]. Free-threshabiliy is also related with tenacious glume (*Tg*) gene, because *Tg* inhibits the expression of *Q* gene. QTL correspond to the *Tg* gene is located on 2D and 2B chromosome. Free-threshing phenotype evolved when mutation transformed *Tg* into *tg*. Therefore free-threshing common bread wheat (*QQ*^5A^*tgtg*^2B^*tgtg*^2D^) and free-threshing durum wheat (*QQ*^5A^*tgtg*^2B^) have mutant alleles at each of the important threshability loci [2,17].

## 4. Exploring Wild Progenitor-Like Emmer Wheat

Wild progenitors of any species generally possess significant genetic diversity which is particularly true for hexaploid wheat. One of the important aspects of modern breeding is to enrich the existing gene pool by introducing important wild genes that were changed, modified or lost during domestication [63]. In the case of wheat, exploring WEM wheat could be great initiative as it played the central role in domestication and it has a rich allelic repertoire for different kinds of agronomic, qualitative, biotic and abiotic stress-related traits [2,17]. Additionally, WEM is fully cross-compatible with durum and bread wheat, so any particular gene of interest can be easily re-introduced into cultivated wheat [14].

The first spikelet of WEM was found and collected by T. Kotschy in 1855 but remained unrecognized. Later, in 1873 it was recognized as WEM by Kornicke, and he published a note on it in 1889 [64]. It was rediscovered in 1906 by agronomist and botanist Aaron Aaronshon in Rosh Pinna and Mount Hermon [65]. Aaronshon first appreciated the importance of WEM for improving tetraploid and hexaploid wheat. At present, emmer wheat is cultivated mainly for human food in small scale that constitutes only 1% of the total wheat area [12]. It has pubescent leaves, persistent enclosed hulls, strong glumes, disarticulated rachis above the spikelet at maturity, pithy culms and short pedicle. Spikes are compacted, laterally flattened narrow, awned and mostly polymorphic for black, green and yellow colored spikes. Spikelets are compressed on the inner side with two florets having red or white, long, thin and pointed end kernel [12].

### 4.1. Geographical Distribution of WEM

WEM grows across the Near Fertile Crescent mostly on basaltic and terra rossa soil but also sometimes on rendzina type soil where winters are comparatively mild [50,66]. According to distribution their accessions can be divided into two different races: The wester race (from Israel, Syria, Lebanon and Jordan) and the central-eastern race (from Turkey, Iraq and Iran); among which the central-eastern one is believed to play the vital role during domestication [17]. The center of distribution and diversity of WEM is observed in the catchment area of upper Jordan Valley and its vicinity, such as the eastern upper Galilee Mountains and in the Golan Heights [31,50]. The populations collected form warm and humid environment (the western Golan, eastern upper Galilee, and north of the Sea of Galilee) are known as the central populations, and includes Yehudiyya, Gamla, Rosh-Pinna, and Tabigha. Populations collected from hot, cold, and xeric peripheries (the northern, eastern, and southern Israeli distribution borders) are known as the marginal steppic-populations, and includes Mt. Hermon, Mt. Gilboa, Mt. Gerizim, Gitit, Kokhav-Hashahar, and J’aba [31]. Generally, robust and early flowering accessions grow on the winter-warm slopes facing the Sea of Galilee, as low as 100 m below sea level; whereas slender and late-flowering accessions grow on cooler and higher elevations, reaching 1600 m from sea level on Mount Hermon [67]. In addition to those habitats, an optimal natural microscale model named “Evolution Canyon (ECI)” was started in 1990 at Lower Nahal Oren, Mount Carmel, Israel with the purpose of long-term research program for addressing the basic evolutionary process of adaptation and speciation. It was expanded to three more additional models, including ECII at Upper Galilee, ECIII at southern Negav desert, and ECIV at the Golan. The ECI covers an area of 7000 m^2^ which harbors around 2500 species from bacteria to mammals and WEM is one of the major model organisms there [68]. The ECI consists of two adjacent slopes separated by 200 m on average: African slope (AS, tropical, xeric, savannoid, south-facing) and European slope (ES, temperate, mesic, forested, north-facing); WEM collected from ES showed a higher level of genetic resistance or defense against biotic stress than that of AS [69,70].

### 4.2. WEM: Genetic Resources with a Great Diversity

WEM occupies the center position of wheat evolution and this long evolutionary process added a lot of variation in multiple important traits that can be grouped as (a) agronomical traits [71]; (b) qualitative traits—protein content [72,73], amino acid composition [74], micronutrient (Zn, Fe) content [73,75]; (c) biotic and abiotic stress-related trait—powdery mildew [70,76], fusarium head blight [77], leaf rust, stem rust, stripe rust [78], yellow rust [79], yellow rust [79], and insects [80]. Genes or QTL for some of those traits have already been identified and mapped from different structured population involving WEM; however, compared with the large existing diversity, most of its variation remains untapped.

### 4.3. Progresses of Using WEM Wheat to Broaden Modern Wheat Gene Pool

**Figure 3:**
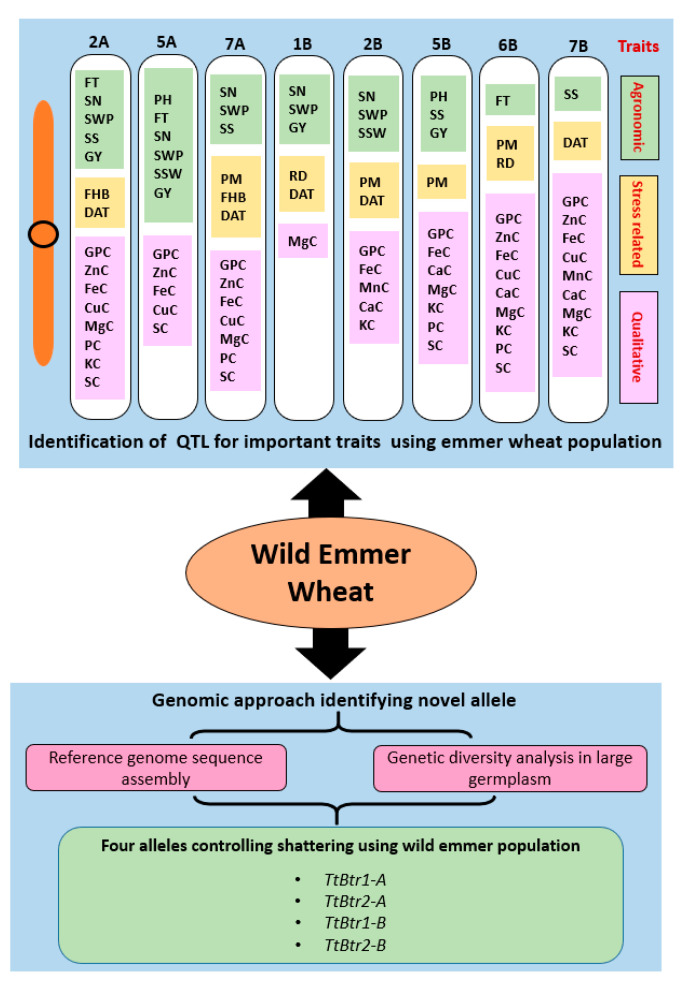
Utilization of WEM wheat through different methods. *Top portion indicates* already identified QTL from WEM for important agronomic, stress-related, and nutrients and quality and *bottom portion indicates* genomic approach used to identify novel allele of spike brittleness from WEM. Note: FT = Flowering time; SN = Seed number; SWP = Seed weight per plant; SS = Seed size; GY = Grain yield; PH = Plant height; SSW = Single spike weight; FHB = Fusarium head blight; PM = Powdery mildew; RD = Rust disease; DAT = Drought adaptive traits; GPC = Grain protein content; ZnC = Zinc content; FeC = Iron content; CuC = Copper content; MgC = Magnesium content; PC = Phosphorus content; KC = Potassium content; SC = Sulphur content; MnC = Manganese content and CaC = Calcium content. TtBtr1-A and TtBtr2-A: loci on chromosome 3A for brittleness; TtBtr1-B and TtBtr2-B: loci on chromosome 3B for brittleness.

That huge genetic diversity of WEM can be used in the breeding of bread and durum wheat to improve the trait of interest. It has been evident that the genetic diversity of modern wheat cultivars has started to increase from 1990 [36]. The reason behind increased diversity is the use of WEM as the donor of novel genes in wheat breeding. In modern wheat breeding the WEM, wheat is being used through three major approaches: (i) Identifying QTL for the traits of interest using genetic mapping; and (ii) identifying novel genes or alleles through genomic approach and integrated them through conventional breeding or transgenic approaches [11,46,81,82,83] (Figure 3).

### 4.4. Identifying QTL/Genes for the Traits of Interest by Genetic Mapping

The widely used approach of utilizing WEM wheat is identifying the QTLs associated with the traits of interest from the WEM germplasms and then integrating that in the bread/durum wheat cultivars through the crossing. An alternative approach other than linkage mapping in the detection of QTLs is association mapping (AM) which is based on searching functional variation in natural populations, aside from the biparental populations, and that does not require linkage maps [84]. It allows researchers to utilize advanced genomics technologies to explore natural populations with a rich diversity of which geneticists and breeders are aware of but has not been utilized much before the genomics era [85].

Research progress in identifying QTL/genes from WEM wheat is discussed here, under the major categories of targeted traits.

#### 4.4.1. Agronomic Traits

Very few agronomic traits, such as plant height, tillering capacity, heading date, spike number, spike weight, seed size, and grain yield have been mapped from WEM [83]. Plant height became a very significant trait for all cereals, especially after the “green revolution” where reduced plant height was achieved, resulting in decreased lodging and increased yield. Two pairs of QTLs have been identified for plant height in 5A and 7B chromosome from WEM using a crossing population between *T. dicoccoides* and *T. durum*. The QTL on chromosome 5A was found responsible for reducing around 9.6–15.2 cm of plant height [46]. Flowering time is another important trait for regional adaptation and yield of wheat [86]. For this trait, four QTL have been mapped on chromosome 2A, 4B, 5A and 6B. Alleles on 2A, 4B, and 6B are responsible for accelerating flowering date whereas that on chromosome 5A for delaying flowering time [46].

Generally, WEM wheat has a very strong tillering capacity. Seven QTL had been detected on five chromosomes, 1B, 2A, 2B, 5A and 7A, which were responsible for spike number. Among them, the 1B and 7A QTL were most important [46]. QTL for spike number on chromosome 1B is homologous with a recessive gene (*tin*) on chromosome 1AS, which is responsible for controlling tiller number [87]. In a cross between WEM and durum wheat, ten QTL were detected for spike weight per plant on 1B, 2A, 5A, and 7A with the 1B QTL being immensely significant [46].

A study on the genetic control of seed size from WEM substitute line in durum wheat background revealed genes with alleles on 1A, 2A, 3A, 4A, 7A, 5B and 7B chromosomes which contributed to seed size variation [45]. Another study mapped eight QTL for seed size on 1B, 2A, 4A, 5A, 5B, 6B, 7A and 7B chromosomes [46]. Similarly, seed number related trait like kernel number per plant (KNP), kernel number per spike (KNS), kernel number per spikelet (KNL), and spikelet number per spike (SLS) are greatly associated with each other and also with total yield. The same study also mapped nine QTL for KNP in six chromosomes, seven QTL for KNS in five chromosomes, seven QTL for KNL in six chromosomes and six QTL for SLS in four chromosomes. Among them, chromosome 5A is highly significant for all four traits [46]. Interestingly, QTL for yield trait was also mapped form the study which identified eight yield QTL on 1B, 2A, 3A, 5A and 5B chromosomes [46]. All these studies demonstrated a high potential in utilizing gene sources in emmer wheat for modern wheat yield improvement.

#### 4.4.2. Biotic Stress-Related Traits

WEM is a good source for resistant genes to a range of biotic stresses, and number of them have been identified and mapped. For example, Powdery mildew is a devastating wheat disease, due to *Blumeria graminis* f. sp. *Tritici* [11,83,88]. Several resistance genes for powdery mildew have been mapped from WEM. *Pm16* was first mapped on chromosome 4A [89] and later on 5BS [90]; *Pm26* on 2BS [91]; *Pm30* on 5BS [92]; *Pm36* on 5BL [93]; *Pm41* on 3BL [94]; *pm42* on 2BS [95]; *MlZec1* on 2BL [96]; *PmG16* and *PmG3M* on 7AL and 6BL, respectively [97]; *MlIW72* on 7A [98]; and *Ml3D232* on 5BL [99].

The rust diseases are also severe and threaten worldwide wheat production as the pathogens have undergone a continuous and rapid virulence evolution [100]. There are three types of rust diseases (i) wheat stem rust, caused by *Puccinia graminis* Pers. F. sp. *Tritici*; (ii) leaf rust, caused by *P. recondite* Rob. Ex Desm. F. sp. *Tritici* and (iii) stripe or yellow rust, *P. striiformis* West. F. sp. *Tritici* [11,83]. Good progress has been made on resistance breeding to rust disease during the past few decades, largely contributed by the identification of several resistance genes from WEM wheat. For stripe rust, some resistance genes have been identified and mapped using cytogenetic or molecular analysis, such as two closely linked genes named *Yr15* [101,102] and *YrH52* [103] on 1BS; *Yr35* on 6BS [104]; and *Yr36* (provides resistance in high temperature) on 6BS [105]. For leaf rust, only one resistance gene, *Lr53* has been mapped from WEM on 6BS using monosomic, telosomic, C-banding and RFLP analysis [104] and this gene is tightly linked with *Yr35*, but independent from *Yr36* [106]. For stem rust, some molecular studies have been conducted in *T. dicoccoides* [78]. A recent study revealed regions on chromosomes 1B, 5A, 5B, 6B and 7B showing resistance against stem rust from a tetraploid wheat population [107].

Fusarium head blight (FHB) is another destructive disease that caused by *Fusarium graminearum* and *F. culmorum* [11,83]. Some variation has been observed in WEM in response to FHB. For example, a QTL, *Qfhs.ndsu-3AS* explained 37% and 55% of the phenotypic and genotypic variances, respectively, in response to FHB [108]. *Qfhs.fcu* is another QTL identified and mapped on 7AL in a WEM accession [109]. On the other hand, some QTL have been identified in 2A of Israeli emmer wheat that increases FHB severity in durum wheat background [110].

#### 4.4.3. Abiotic Stress-Related Traits

WEM has the ability to grow in dry and saline soil in the Middle East, and it was the first indication that WEM can be a good source of genes for abiotic stress tolerance [83]. The uptake rate of Na^+^ was lower in some accessions of WEM from Israel compared to cultivated durum when seven days seedlings were exposed to 1mM NaCl solution for two days [74]. Some other accessions even survived in 175 to 250 mM NaCl solution until maturity resulting loss in average dry weight per plant only around 35% [111]; whereas almost similar treatment results in 90% loss in average weight per plant for durum cultivar [112]. Therefore, WEM wheat could be a great source for salinity tolerance genes, though no specific QTL/gene has been identified yet. Another major abiotic stress that causes massive yield loss is drought which could be reduced by utilizing the drought-tolerant novel genes from WEM wheat. A study on mapping population comprising recombinant inbreed line from a cross between durum and emmer detected 22 QTL for drought susceptibility index [113]. Furthermore, a transcriptome analysis on WEM identified a number of differentially regulated transcripts between drought resistant and susceptible genotypes and of which 221 transcripts were highly expressed in drought-resistant genotypes [114]. In another study on WEM, a gene for drought-inducible putative membrane protein from root tissue was cloned, characterized and named as *TdicTMPIT1*. This gene was upregulated when exposed to drought stress in drought-tolerant WEM accessions, but remained unchanged in drought susceptible accessions or in cultivated durum [115]. All these genes have the potential to be a source of the drought-resistant gene for improvement of wheat genotypes.

#### 4.4.4. Quality and Nutritional Traits

WEM wheat has several QTL/genes for various qualitative traits like grain protein content (GPC) and micronutrient (Zn, Fe, and Mn) content. GPC is one of the important attributes to determine the nutritional value and quality of bread products [11,83]. WEM has been shown to have higher GPC content (20–24%) than cultivated wheat [116]. The first QTL for high GPC was detected in WEM accession FA-15–3 (Avivi 1978) which was later mapped as *Gpc-B1* on chromosome 6BS [117,118]. Cloning and annotation of this gene revealed it as a NAC transcription factor that controls the remobilization of nutrient into the sink during leaf senescence [73]. Besides, three QTL controlling GPC on chromosome 5B [119] and another three GPC QTL on chromosome 2AS, 6AS, and 7BL have been identified and mapped in WEM accessions [120]. Furthermore, a study on 23 substitutions lines based on two accessions of WEM (PI 481521 and PI 478742) revealed a set of novel genes for high GPC on chromosome 1A, 2A, 5B and 7B of PI 481521 and 7A, 5B, 6B and 7B on PI 478742 [121]. In addition to GPC, WEM has a highly variable seed storage protein compositions with a high number of novel alleles. A number of studies on high molecular weight (HMW) glutenin loci (*Glu-1A*, *Glu1B*, *Glu1D*), avenin like protein (*ALP*) and monomeric alpha-amylase inhibitors (*WMAI*) revealed a considerable number of alleles for those genes [31,122,123,124,125,126,127,128]. Among the HMW glutenin subunits, only one gene *1By18* is the consequence of gene mutation from *1By8* through the formation of hexaploid wheat, and the rest are on their original forms as of the ancestors [125]. Since many seed storage protein alleles from WEM have not entered into hexaploid wheat, a high potential exists to broaden the seed storage protein gene pool for breeding, through exploring and utilizing the alleles of the WEM.

WEM has considerable variation in micronutrient content which makes it valuable for improving mineral concentrations in cultivated wheat. In a study, zinc and iron concentration was the highest in 6A, 6B and 5B WEM chromosome substitution lines among 825 accessions of worldwide wheat collections [75]. In another study, a total of 82 QTL for 10 minerals were mapped in a recombinant inbred line (RIL) population developed from a durum X WEM wheat cross. A significant positive association was found between GPC and nutrient (Zn, Fe, and Cu) content, suggesting a possible overlap of the respective QTL, such as QTL on 2A, 5A, 6B, and 7A chromosomes [129]. Not only micronutrients (Zn, Fe, Cu, and Mn), but the macronutrient content (Ca, Mg, K, P and S) of WEM also has a great diversity. A study on 154 genotypes comprising WEM along with diverse wheat varieties demonstrated that WEM accessions had a wide genetic variation for all nutrient contents, with the Zn, Fe, and protein contents being two-fold larger than these of cultivated varieties [14].

### 4.5. Genomic Approach Using WEM Genotypes for Novel Allele Identification

The rapid advancement of several genomic approaches made it possible to reveal the molecular mechanisms underlying domestication and evolution, including: (i) Reference genome sequence assembly for domesticated crops and wild relatives; (ii) genomic characterization to survey sequence diversity in large germplasm collections; and (iii) application of novel methodologies, such as population genetics, epigenomics, and gene editing [130]. Reference genome sequence assembly means alignment and merging of DNA fragment from a sequenced genome to reconstruct the original one through producing pseudomolecules which provides a way to identify novel beneficial allele by a genomic comparison between domesticated crop and their progenitors [82,131]. To analyze genetic diversity form a large germplasm collection, several high-throughput sequencing-based methods are now available, such as whole genome sequencing, exome capture, RNAseq and genotyping by sequencing [130]. Till date, only one durum wheat accessions “Svevo” was used for reference assembly describing 10.45 Gb assembly of 14 chromosomes of durum wheat [82]. Similarly, only one WEM accessions “Zavitan” was selected for reference assembly which reported a 10.1 Gb assembly corresponding to 14 chromosomes of WEM along with gene content, genome architecture and genetic diversity analyses [81]. A Svevo vs. Zavitan comparison and a survey for genetic diversity upon a collection of global tetraploid wheat lines were performed and showed that the regions bearing signature for domestication and evolution were well distributed over the genome with diversity reduction in the pericentric region [81,82]. This kind of assembly has been found very useful for identifying genes using a population derived from a cross between two dissimilar parents. Since WEM wheat is the direct progenitor of modern wheat with wide diversity, more WEM accessions should be sequenced and assembled to identify unknown allele of varied interest.

## 5. Conclusions

This review focused on three important aspects of wheat genetics and evolution: (i) The long wheat evolutionary process, including hybridization, domestication, polyploidization, and mutation; (ii) genome modifications occurred during evolution, including gene loss that might have important roles for wheat improvement; and (iii) utilization of WEM for identifying novel genes that has not entered into bread or durum wheat. It demonstrates that utilizing the novel gene alleles in WEM is an efficient and feasible way for a wide range of trait enhancements in modern wheat breeding. This is largely due to the WEM’s crossability advantages over other wild species, making it possible for the beneficial genes or alleles from WEM to be directly incorporated into bread and durum wheat. There are also wild relatives of modern wheat that have a high level of gene variations, but most of those species are not cross compatible with bread or durum wheat. Comparing with the existing huge untapped genetic variation, the number of genes already been cloned from WEM is rather limited. Currently, an imminent task is to identify and clone more important genes from WEM that confer agronomic, qualitative, biotic and abiotic stress-related traits. To achieve this, QTL or controlling genetic factors in WEM for these traits must be identified first, which has long been difficult due to the exceptional variable traits, as well as the lodging and shattering problem of WEM, which make the phenotyping rather challenging. However, the development of chromosome arm substitution lines has made it possible to fast identify genes in WEM [128]. Meanwhile, the emerging high throughput phenotyping platforms have eased up the QTL and controlling gene identification through genome wide association mapping. Additionally, the complete genome sequence is now available for both bread wheat and WEM, which ultimately speeds up the gene identification process. As a summary, utilizing novel alleles of WEM in modern wheat breeding has a high potential in breeding cultivars for sustainable wheat production under the ever-changing global farming environment.

## Figures and Tables

**Table 1:** Important traits were considered for domestication and the responsible genes or QTLs with their location.

Trait	Description of the Traits in Relation to Domestication	Gene Name	QTL Position
Brittle rachis [42,43]	This trait is agriculturally deleterious, and thus, the transformation of brittle rachis to non-*Br* is perhaps the first symbol of domestication in wheat.	*Brittle rachis* (*Br1*, *Br2* and *Br3)*	3DS, 3AS and 3BS
Glume tenacity [43,44]	The wild wheat floret is wrapped by tough glumes that make spikes difficult to thresh, whereas cultivated wheats have soft glumes and are free-threshing.	*Tough glumed* (*Tg1*) *soft glumes* (*sog*)	2A, 2B, 2D, 5A, 6A, 6D and 7B
Free-threshing [1]	The *Q* gene is a major domestication gene conferring spike shape and threshability in wheat. Increased transcription of *Q* was associated with spike compactness and reduced plant height.	*Threshability gene* (*Q gene*)	5AL
Seed size/weight [40,45,46]	Increase in seed size or weight took place before the evolution of non-shattering ears. The trait is under complex polygenic control for all domesticated cereals.	-	1A, 1B, 2A, 3A, 3B, 4A, 4B, 5A, 5B, 6A, 6B, 7A, 7B
Seed shape [40]	Grain shape is an important attribute for ensuring market quality. Domestication has transformed long and thin primitive grains to wider and shorter modern grain.	*Grain size* (*GS3*) *grain weight* (*GW2*) *seed width* (*SW5*)	1A, 3A, 4B, 5A, 6A
Flowering time [1,40,46]	Domestication involved selection of spring wheat that lack of vernalization and specific photoperiod requirement. The wild allele on 5A of *T. dicoccoides*, responsible for late-flowering, is similar to the *VRN1* gene and also present in a collinear position with *Ppd* genes.	-	2A, 4B, 5A, 6B
Grain yield [1,47,48]	Yield was considered to be one of the important traits for domestication which minimize the labor input and land needs. Yield QTL is overlapped with QTL for other traits.	-	1B, 2A, 3A, 4A, 5A, 5B,
Plant height [1,46]	Though reduced plant height is desired for modern wheat breeding, tall mutants with higher biomass and yielding potential were historically selected.	-	5A, 7B
Spike number/plant [1,46]	Spike number is strongly correlated with tillering capacity. A single recessive gene (*tin*) on 5A, controlling tiller number is assumed to be homologous with QTL for spike number on 1B of *T. dicoccoides*.	-	1B, 2A, 2B, 5A, 7A
Spike weight/plant [1,46]	These all traits are highly correlated with each other and also with grain yield. QTL for these yield-related traits were found in different chromosomes, among them 5A, 2A and 1B had the most significant role in domestication.	-	1B, 2A, 3A, 5A, 5B, 7A
Single spike weigh [1,46]		-	1B, 2A, 3A, 5A
Kernel number/plant [1,46]		-	1B, 2A, 3A, 5A, 5B, 7A
Kernel number/spike [1,46]		-	1B, 2A, 3A, 5A, 6B
Kernel number/spikelet [1,46]		-	1B, 2A, 3A, 5A, 5B, 7B
Spikelet number/spike [1,46]		-	1B, 2A, 5A, 6B
